# Spontaneous Coronary Artery Dissection: A Case Series of 9 Patients With Literature Review

**DOI:** 10.1177/2324709618770479

**Published:** 2018-04-18

**Authors:** Ali Raza Ghani, Faisal Inayat, Nouman Safdar Ali, Reema Anjum, Michael Viray, Arsalan Talib Hashmi, Iqra Riaz, Bruce D. Klugherz, Hafeez Ul Hassan Virk

**Affiliations:** 1 Abington Hospital – Jefferson Health, Abington, PA, USA; 2 Allama Iqbal Medical College, Lahore, Pakistan; 3 Einstein Medical Center, Philadelphia, PA, USA; 4 Maimonides Medical Center, Brooklyn, NY, USA

**Keywords:** spontaneous coronary artery dissection, acute coronary syndrome, myocardial infarction, fibromuscular dysplasia, pregnancy-associated spontaneous coronary artery dissection, diagnosis, management

## Abstract

Spontaneous coronary artery dissection is an increasingly recognized nonatherosclerotic cause of acute coronary syndrome. Reports regarding the prognosis and natural history of this disease are limited. In addition to the diagnostic difficulty, this condition poses a significant therapeutic challenge due to the lack of specific management guidelines. We present here a case series of 9 patients with spontaneous coronary artery dissection. Additionally, this article reviews the incidence, clinical characteristics, risk factors, diagnostic modalities, therapeutic approaches, and patterns of recurrence in patients with spontaneous coronary artery dissection.

## Introduction

Spontaneous coronary artery dissection (SCAD) is an underdiagnosed clinicopathologic entity. It is characterized by a tear in the coronary arterial wall that is nontraumatic and noniatrogenic with no specific secondary etiology of coronary dissection. As a nonatherosclerotic cause of acute coronary syndrome (ACS), SCAD can be life threatening due to its sudden onset, fulminant progression, and the dilemma associated with the diagnosis.

The clinical presentation is varied. A study of a large cohort of 196 patients with SCAD (women, n = 178) reported that 96% of patients presented with chest discomfort. Other presenting symptoms included arm pain (49.5%), neck pain (22.1%), nausea or vomiting (23.4%), diaphoresis (20.9%), dyspnea (19.3%), and back pain (12.2%).^1^ Furthermore, 8.1% of patients developed ventricular tachycardia and/or ventricular fibrillation while 1% presented with cardiac arrest.^1^ Although these presentations are similar to the initial symptoms of atherosclerotic causes of ACS, women with atherosclerotic heart episodes are far less likely to present with chest pain as compared with the women with SCAD.^1^ ST-segment elevation myocardial infarction (STEMI) was identified in 24% of the patients and the rest had non-STEMI (NSTEMI).^1^

The pathogenesis of SCAD remains controversial and a number of mechanisms have been proposed. It is hypothesized that a spontaneous intimal tear results in bleeding into the coronary arterial wall leading to hematoma development that culminates in blockage of the true arterial lumen.^2^ Alternatively, it is speculated that an initial vasa vasorum disruption inside the coronary arterial media may lead to hemorrhage and subsequent hematoma formation with no connection to the arterial lumen. Myocardial ischemia ensues following compression of the coronary arterial wall.^3^ Furthermore, a restricted role of genetics in the pathophysiology of SCAD with some component of recessive inheritance patterns in sporadic cases has also been implicated.^4^ The pathogenesis, however, is still lacking precision, prompting further investigation.

This article describes 9 patients with SCAD. It further highlights that physicians should be particularly vigilant for atypical presentations of this disease. It should be included among differentials, especially in young females presenting with ACS, in the absence of notable risk factors for cardiovascular illness.

## Case Series

### Case 1

**Figure 1.**
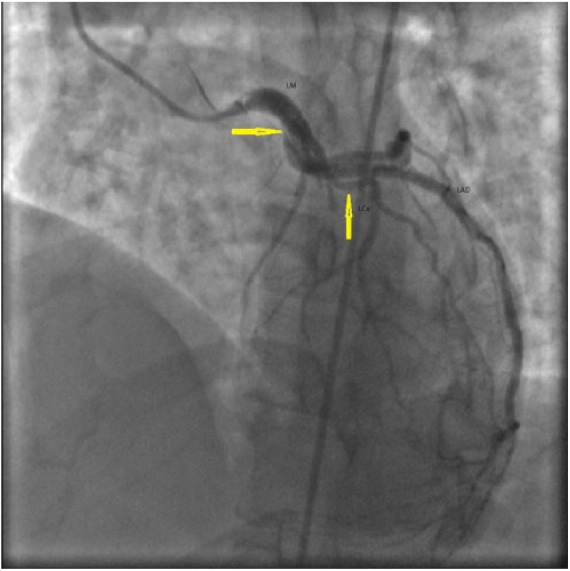
Case 1. Right anterior oblique (RAO) cranial image of left coronary angiography showing spontaneous coronary artery dissection of left main (LM) and left anterior descending (LAD) artery (arrows).

A 41-year-old female with a past medical history of hypothyroidism and Prinzmetal’s angina presented with worsening typical chest pain for 1 day. She was 10 weeks postpartum. Her pain was unresponsive to nitroglycerin and aspirin. Electrocardiogram showed T-wave inversions in anterolateral leads. She was taken to the cardiac catheterization laboratory and was found to have SCAD of the left main (LM), left anterior descending (LAD), and left circumflex arteries (LCx; Figure 1). She was managed with emergent coronary artery bypass graft (CABG) of 2 vessels, including, left internal mammary artery to the LAD and saphenous vein graft (SVG) to the ramus intermedius artery. Her subsequent recovery was uneventful and she was asymptomatic with normal echocardiogram at the 6-month follow-up.

### Case 2

**Figure 2.**
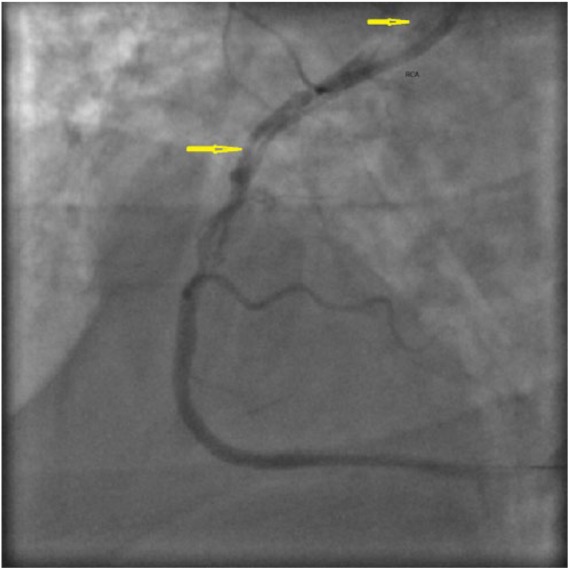
Case 2. Right anterior oblique (RAO) cranial view of right coronary angiography showing spontaneous coronary artery dissection of right coronary artery (RCA) (arrows).

A 51-year-old male with a history of SCAD presented to our medical Center with refractory angina and decreased exercise tolerance for 2 days. Electrocardiogram showed nonspecific ST-T wave changes with initial troponin level of 0.8 ng/mL (normal <0.01 ng/mL). He was taken to the cardiac catheterization laboratory where coronary angiography showed dissection of both the LAD and the right coronary artery (RCA; Figure 2). He was managed with the deployment of 2 overlapping drug-eluting stents with good angiographic results in the RCA. In his LAD, he had a residual dissection with an angiographically determined good flow. At the 3-month follow-up, he showed recovery of his exercise tolerance and no further episodes of angina.

### Case 3

**Figure 3.**
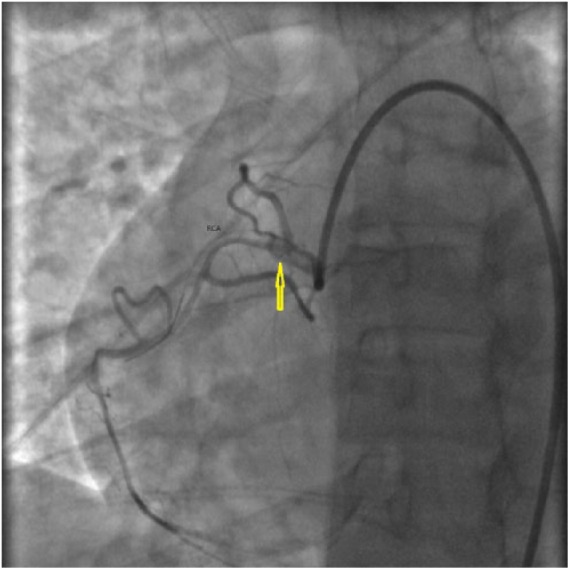
Case 3. Right anterior oblique (RAO) cranial view of right coronary angiography showing spontaneous coronary artery dissection of proximal to mid right coronary artery (RCA) (arrows).

A 34-year-old African American female with a recent Cesarean section presented with sudden-onset chest pain for 15 minutes. On admission, electrocardiogram showed ST-segment elevations in V4, V5, and V6. Cardiac catheterization was performed. It revealed a long dissection of the LAD, originating just distal to the ostium and extending up to the mid portion. Thrombolysis in myocardial infarction (TIMI) grade 3 flow (normal flow) was noted. She was initially managed with medical therapy. Two days later, she had recurrent chest pain. Electrocardiogram changes were consistent with ischemia. Repeat catheterization showed stable LAD dissection with new RCA dissection with TIMI grade 1 flow (incomplete filling of distal coronary artery; Figure 3). She underwent an emergent 2-vessel CABG (SVG to LAD and SVG to RCA). Her postoperative period was uneventful. She developed peripartum cardiomyopathy after 3 months but had recovered left ventricular systolic function at the 6-month follow-up.

### Case 4

A 45-year-old female, professional marathon runner, with a history of Raynaud’s phenomenon, migraines, gastroesophageal reflux disease, and serum-positive HLA-B27, developed a sudden-onset chest pain while riding her bicycle. The pain lasted 2 hours, throughout the duration of exercise, and was noted to radiate to her back and jaw. In the emergency room, the electrocardiogram showed anterior wall myocardial infarction (MI) with positive serum troponin of 0.15 ng/mL (normal <0.01 ng/mL). Cardiac catheterization ruled out coronary atherosclerotic disease. However, the LM had a long SCAD that was extending to the mid LAD. The blood supply distal to the lesion was compromised. It was successfully stented with 2 bare metal stents. At the 3-month follow-up, she had an uneventful recovery with partial recommencement of her strenuous physical activity.

### Case 5

**Figure 4.**
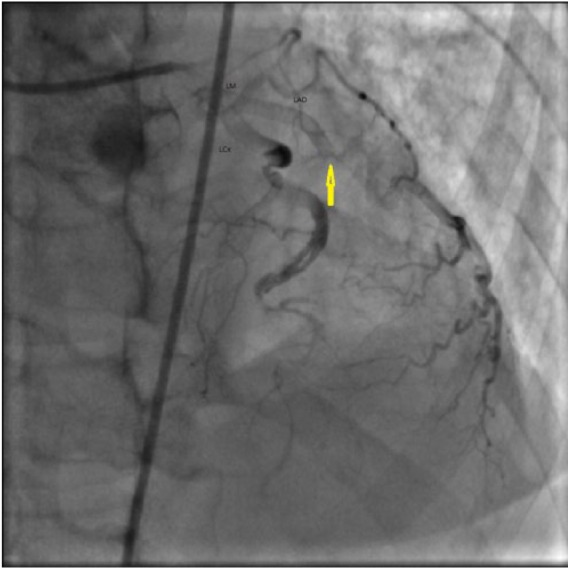
Case 5. Right anterior oblique (RAO) cranial view of left coronary angiography showing spontaneous coronary artery dissection of mid-left anterior descending (LAD) artery (arrows).

A 49-year-old female with stage IV sarcoidosis presented to our hospital with chest pain and shortness of breath. Electrocardiogram showed STEMI in the anterolateral leads. Emergent catheterization revealed SCAD in the mid LAD with an unsuccessful wiring (Figure 4). The patient was managed conservatively. Later, her hospital course was complicated by a left ventricular thrombus and an embolic stroke requiring anticoagulation. She was discharged to rehabilitation center where she stayed for a period of 6 weeks. At the follow-up in the outpatient clinic, the patient had no residual deficits of recent stroke and had no angina. Over the next year, she had worsening pulmonary hypertension secondary to her sarcoidosis and was deemed a suitable candidate for a heart and lung transplantation.

### Case 6

**Figure 5.**
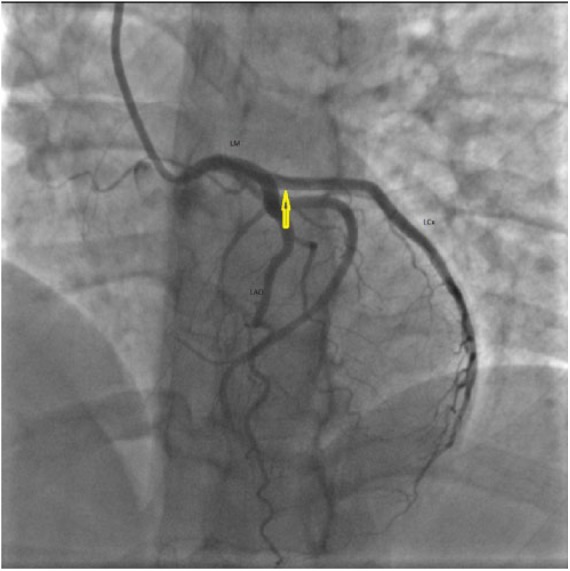
Case 6. Left anterior oblique (LAO) cranial view of left coronary angiography showing spontaneous coronary artery dissection of left circumflex artery (LCx) (arrows).

A 56-year-old female developed sudden-onset, sharp chest pain that led to syncope. On admission, electrocardiogram showed NSTEMI with a troponin level of 4.5 ng/mL (normal <0.01 ng/mL). Left heart catheterization showed a SCAD involving the LCx with TIMI grade 3 distal flow (Figure 5). While receiving cardiac catheterization, she developed an acute stroke with left-sided visual field deficit and received intravenous tissue plasminogen activator with complete resolution. She was managed conservatively with medical therapy for NSTEMI. She remained compliant with her medications. Follow-up catheterization due to persistent chest pains showed healed LCx dissection with no atherosclerotic disease in the rest of her coronary arteries. She was started on nifedipine for possible diagnosis of Prinzmetal’s angina (variant angina). Her symptoms improved drastically on subsequent follow-up visits.

### Case 7

**Figure 6.**
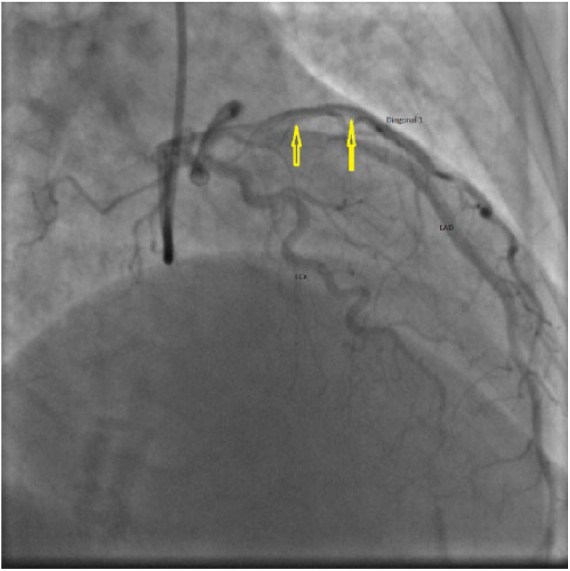
Case 7. Right anterior oblique (RAO) cranial view of left coronary angiography showing spontaneous coronary artery dissection of first diagonal branch (D1) of left anterior descending (LAD) artery (arrows).

A 60-year-old female presented to our medical institution with chest pain lasting 15 minutes. Electrocardiogram showed a new-onset right bundle branch block and she was found to have a troponin of 0.24 ng/mL (normal <0.01 ng/mL). Coronary angiogram showed an abnormality in the diagonal branch of the LAD (mid D1 radiolucency) concerning for thrombus versus dissection (Figure 6). Optical coherence tomography was performed to distinguish the lesion. Therein, a SCAD was noted, which was managed with drug-eluting stent placement. Final angiography displayed no evidence of thrombosis, distal embolization, or further dissection. She remained compliant with her dual antiplatelet therapy for 8 months. Subsequently, she experienced a major gastrointestinal bleeding after which clopidogrel (Plavix) was discontinued. She remained symptom-free on follow-up visits.

### Case 8

**Figure 7.**
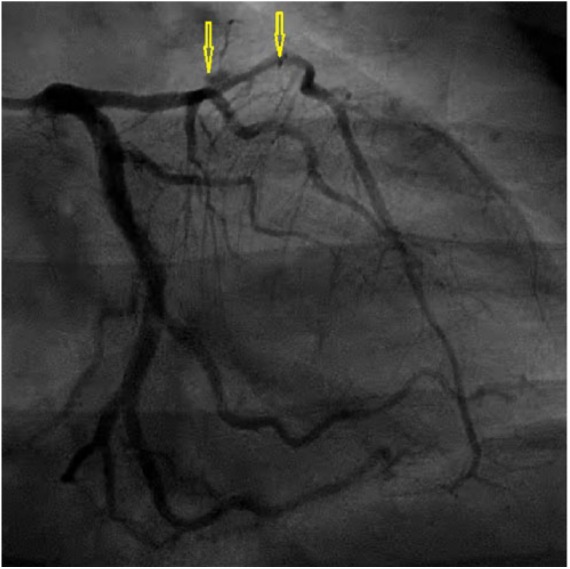
Case 8. Right anterior oblique (RAO) caudal view of left coronary angiogram showing spontaneous coronary artery dissection of mid left anterior descending (LAD) artery with intramural hematoma (arrows).

A 34-year-old female who was 38 weeks pregnant presented with atypical chest pain for 1 day. Electrocardiogram showed ST-segment elevations in the anterolateral leads with a negative first troponin level. Coronary angiogram showed SCAD of the mid portion of the LAD with large intramural hematoma compromising the blood flow to the first and second diagonal branches of the LAD with TIMI grade 3 flow into distal vessel (Figure 7). No intervention was done and she was admitted to the cardiac care unit where a conservative approach was adopted. After undergoing Cesarean section, repeat coronary catheterization was performed. It showed no evidence of intramural hematoma and a healed LAD dissection.

### Case 9

**Figure 8.**
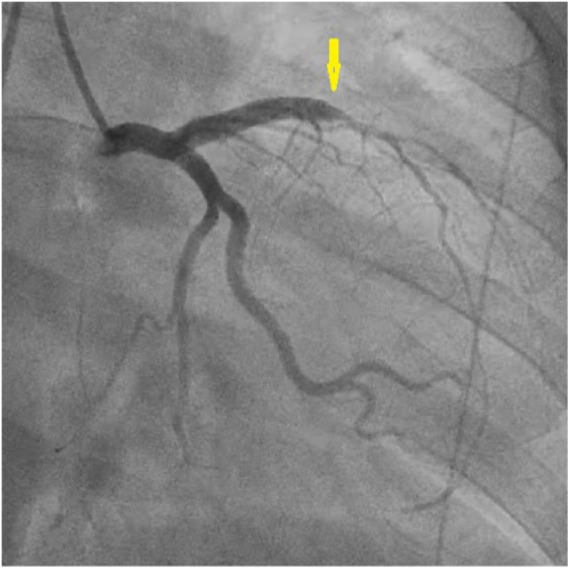
Case 9. Right anterior oblique (RAO) caudal view of left coronary angiography showing spontaneous coronary artery dissection of proximal left anterior descending (LAD) artery with compromised blood flow to diagonal branches (arrows).

A 36-year-old female who was 6-days postpartum presented with typical chest pain radiating to her left arm for 1 hour. Electrocardiogram showed ST-segment elevations in leads V2 and V3 and ST depressions in the inferior leads. Emergent catheterization showed a dissection of the proximal LAD after the first 2 septal perforators with evidence of luminal compromise by a hematoma (Figure 8). Conservative management was planned and she was started on dual antiplatelet therapy. Follow-up catheterization after 6 weeks showed no progression of the LAD dissection with resolution of the hematoma. She remained asymptomatic on follow-up visits.

## Discussion

SCAD was first described by Pretty in 1931.^5^ Since then, several cases have been reported worldwide. Although it has been identified as a distinct clinical entity, an accurate prevalence is unknown. The incidence in patients with ACS was 2/1000 as reported by the Denmark Heart Registry.^6^ In a prospective evaluation, 0.3% of the patients with stable angina, 2.9% with acute MI, and 4.2% with post-MI angina developed SCAD.^7^ This disease has a predilection to develop into ACS in young, apparently healthy females. Recently, a contemporary case series showed the prevalence of SCAD in the range of 22% to 35% in women less than 50 years of age, with the major disease burden concentrated in middle-aged women.^8^ In this study, the mean age for female patients was 44 years. The data on patient demographics, comorbid conditions, clinical presentation, distribution of SCAD lesions, management, and clinical outcomes in participants of our study are summarized in Table 1.

**Table 1.** Patient demographics, risk factors, clinical presentations, the respective involvement of coronary artery, and management in participants of the present study.

Baseline Characteristics	Male	Female
Mean age (years)	51 (n = 1)	44 (n = 8)
Diabetes	0	0
Hypertension	0	0
Hyperlipidemia	0	0
Smoking	0	0
Oral contraceptive use	NA	0
Pregnancy	NA	3
FMD	0	0
Presentation		
STEMI	0	6
NSTEMI	0	1
Refractory chest pains	1	0
Others	1	0
Distribution		
LAD	1	7
LM		1
RCA	2 (1 + 1)	1
LCx	0	5
Multivessel disease	1	2
Management		
Medical therapy	0	4
PCI	1	2
CABG	0	2
Clinical outcome		
Recovered	9	
Died	0	

Abbreviations: FMD, fibromuscular dysplasia; NA, not applicable; STEMI, ST-segment elevation myocardial infarction; NSTEMI, non–ST-segment elevation myocardial infarction; LAD, left anterior descending; LM, left main artery; RCA, right coronary artery; LCx, left circumflex; PCI, primary coronary intervention; CABG, coronary artery bypass graft.

The risk factors for SCAD have been recently studied in detail. In one study that evaluated 327 patients with SCAD, fibromuscular dysplasia (FMD) was present in 62.7% of the patients.^9^ However, there were only 3.4% of FMD patients who had been diagnosed with SCAD according to the large FMD registry data of patients from the United States.^10^ It is notable that up to 86% of SCAD cases have either FMD or another underlying arterial disorder.^11^ FMD is poorly understood and its diagnosis is often delayed for several years. Our case series is unique in this regard as none of the patients recruited in our study had FMD. Furthermore, the patients did not have a history of comorbid conditions, such as diabetes, hypertension, or hyperlipidemia. All of them were nonsmokers and none of the female patients were on oral contraception at the time of admission.

Pregnancy is another huge risk factor for SCAD, with the most frequent presentation of ACS in the postpartum period.^12,13^ Stress has also been commonly linked with the development of SCAD. In one study, 57% of the patients who were dealing with either mental or physical stress developed SCAD. Physical activity in the form of exertion and mental stress in the form of emotional distress were associated with increased catecholamines and a higher risk of SCAD.^11^ Coronary artery tortuosity on angiography is also shown to have a strong association with SCAD. A case-control study reported 61% higher prevalence of coronary tortuosity and a 2-point higher tortuosity score in the SCAD group as compared with the control.^14^ Other risk factors include connective tissue disorders, multiparity, hormonal therapy, systemic inflammatory disease, and hypertension.^9,15,16^ In this study, 8 of the 9 patients were females and 3 of them were either pregnant or postpartum at the time of admission to our hospital for SCAD.

This disease has been categorized into 3 main types on the basis of angiographic features. Type 1 is the typical disease presentation that is characterized by multiple coronary artery lumens associated with the longitudinal filling defect. Type 2 is the most commonly encountered entity where patients develop diffuse vessel stenosis. Type 3 disease is usually marked with focal vessel stenosis. Both type 2 and type 3 show elusive changes in the caliber, signifying a hematoma in the wall of the involved vessel.^17^ Intravascular imaging is considered mandatory to confirm type 3 SCAD as it cannot be differentiated from atherosclerosis.^18^

Coronary angiography is the first-line investigation for SCAD. Although it yields the diagnosis in most of the cases, this condition may pose a significant diagnostic challenge in difficult-to-diagnose patients or when the concerned physicians are not experts in their field. The use of intravascular ultrasound is becoming increasingly popular because of the advantage of better visualization, correct placement of guide wire, and optimal deployment of coronary stents.^19^ Intravascular ultrasound effectively differentiates between the true and false lumens. In cases of SCAD with intimal tears, it has the added benefit of identifying the intimal tear length and morphology. In cases without intimal tears, it helps in confirming the length of intramural hematoma as well as morphology.^20^ Optical coherence tomography is another light-based imaging tool that can be employed in patients with SCAD for better visualization and demarcation of intramural hematoma.^21^

Medical treatment of SCAD is highly individualized. It has a weak scientific evidence base due to the lack of randomized controlled trials. A consensus has been developed on using dual antiplatelet therapy for a limited duration. Beta-blockers are frequently employed, and a recent study found a significant role of β-blockers in the prevention of recurrent SCAD.^9^ Angiotensin-converting enzyme inhibitors are used in patients with left ventricular dysfunction. The use of statins in SCAD is avoided by some clinicians and it is a matter of debate.^22^ The most common outcome in stable patients is the resolution of SCAD within 3 months, which can be achieved with supervised conservative management.^23^

The question of revascularization is unsettled in patients with SCAD. If revascularization is required, percutaneous coronary intervention (PCI) is preferred to CABG. PCI in SCAD patients has been known to have a higher complication rate when compared with PCI in patients with atherosclerosis.^24^ Patients with unstable presenting complaints have a higher probability of undergoing revascularization.^1^ Pregnancy-associated SCAD may cause the extension of dissection in approximately half of all patients and PCI is successful in nearly a quarter of patients.^16,25^ Post-PCI patients who receive a stent or have elevated blood pressure may be initiated on aspirin, heparin, β-blockers, and/or nitrates. SCAD involves the mid to distal LAD artery in majority of the cases, and the proximal LAD and the LM coronary artery in pregnancy-related SCAD patients.^16,25^ However, therapeutic PCI is not employed in patients involving LM and ostial LAD arteries, and CABG is the treatment of choice in these cases.^22,26,27^ However, there are chances of connecting false lumen in patients with SCAD undergoing CABG. Therefore, it necessitates extra care to properly identify the lesion.

Recurrence of SCAD is a major contributor to morbidity. Of 327 patients from Vancouver, Canada, 100% had early survival to discharge.^9^ After a median follow-up of 3.1 years, recurrent SCAD developed in 10.4% of these patients. Multivariate analysis showed that hypertension was associated with a higher risk of recurrent SCAD with a hazard ratio of 2.46 (95% confidence interval = 1.23-4.93) and a highly significant *P* value of .011. On the other hand, the use of β-blockers decreased the risk of recurrence by two thirds with a hazard ratio of 0.39 (*P* = .004). Generally, female sex and absence of early treatment were the strongest predictors of adverse clinical outcomes.^28^ In the present study, 4 patients had stable disease who were managed with medical therapy. Three patients underwent PCI and 2 received CABG. The recovery was uneventful in all patients with no signs of recurrence on subsequent follow-ups.

## Conclusion

SCAD is an underrecognized syndrome that predominantly affects young females. Clinical features are usually consistent with ACS. In terms of management, standard guidelines are nonexistent for patients with SCAD. In hemodynamically stable patients, conservative therapy maybe considered the mainstay of disease management. Unstable patients can ultimately be treated with PCI or CABG. The risk of recurrence is significantly high in the few years after the primary event and a regular imaging follow-up is warranted. Future research in stratification of the long-term cardiovascular outcomes and determination of an accurate risk for recurrence will be valuable for early diagnosis and management in patients with SCAD.
